# Sense of space: Tactile sense for exploratory behavior of roots

**DOI:** 10.1080/19420889.2018.1440881

**Published:** 2018-03-26

**Authors:** Ken Yokawa, František Baluška

**Affiliations:** a Center for Bioscience Research and Education, Utsunomiya University, Tochigi, Japan; b IZMB, University of Bonn, Bonn, Germany

**Keywords:** plant root, root tropism, spatial exploration, exploring behavior

## Abstract

In soil, plant roots grow in heterogeneous environments. Plant roots are always facing the difficulty of searching effectively the patchy natural resources, such as water, oxygen, ions and mineral nutrition. Numerous studies reported that root apex navigation enables roots to explore complex environments. In this short communication, we characterize how growing maize roots explore narrow space available with two experimental settings: tactile exploration of narrow glass tube and circumnutation in free space. We also discuss root growth in the soil in terms of foraging behavior guided by the sensory root apex.

Unlike comfortable well-equipped and conditioned laboratories, natural environments fluctuate drastically, changing resources available for survival of living organisms. While animals can move away from challenging environments and search for food and shelter, sessile plants can only alter their physiological processes and conditions to cope with variable circumstances such as light, temperature, humidity and so on. Although plants seem to bear harsh environment passively, plant organs (especially roots) have the ability to direct the growth ‘positively’ for survival, which is called tropic behavior. In the pioneering studies, Charles and Francis Darwin firstly demonstrated that root tip is a sensory organ which controls the direction of root growth [1]. It is quite important ability for plant roots to grow towards nutrition enriched soil patches and to avoid unfavorable and toxic patches. If plant roots would only keep waiting until water and nutrient will approach root surfaces, they would not win the fierce natural competition among other species because resources are very limited in nature. Therefore, strategy for foraging resources is highly demanded. Generally, many living organisms use senses, for example olfactory, visual, tactile senses to explore and exploit their environment. Plants can sense light [2], gravity, sound and vibrations [3], as well as volatiles [4]. Roots modulate their growth direction and development in response to nature, intensity or direction of these cues. Based on these multiple tropic behaviors, roots can actively reach nutritional rich patches in the soil, or avoid toxic and depleted ones. The complex root systems, which can reach immense complexities and sizes, represent a trajectory of these searching actions; which started with the seed germination at the soil surface and continued by exploratory root growth in the underground.

Among senses described above, tactile sensation is essential to estimate the texture and the distance to object for many species. Do plants use this sense to get the information of space available in soil? It can be expected that roots are exploring narrow spaces of the soil in darkness by touching soil/rock surfaces. Underground is heterogeneous and challenging environment with limited space due to dense soil particles, stones and rocks; all restricting free growth of roots. Although it is well-known that plant root responds to touch stimuli via so-called mechanical sensation [3,5], there is almost no investigation and discussion about the possible space explorations via growing roots. Here we describe root behavior imposed on growing maize roots by challenging them via narrow glass tubes. Roots are obviously able to sense the available space and even to accomplish rather difficult U-turn movements if stressed by light.

## Root exploration of narrow spaces: Following paths of soil worms?

It is known that root growth is promoted when earthworms or arthropods were inoculated to the soil, possibly due to small pores drilled by those invertebrates in compacted soil [6]. It is also known that roots utilize animal-made pores or cracks allowing unhampered elongation of roots in soil [7]. Very dense distribution of plant roots was found in the soil region with macropores [8]. These finding suggest that roots prefer to grow in the cracks or pores once these are available to them.

**Figure 1.**
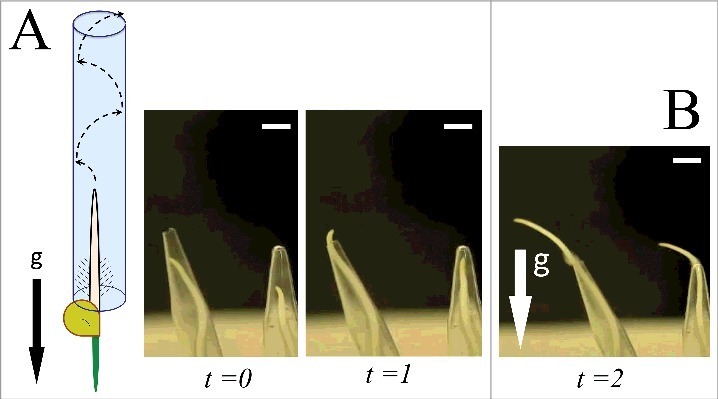
Maize roots growing in upside-down capillary. A. Roots are growing upwards by touching the glass surface. B. Time-lapse pictures show roots growing inside the glass capillary and going out into open space (see Supplementary movie 1). Roots show this upward-growth only in darkness. Scale bar indicates 5 mm.

Figure 1 shows the schematic illustration that maize roots grow in narrow capillary in up-side-down way. Roots keep growing against gravity vector in darkness, whereas root apices immediately U-turn by illumination from above [9]. It implies important issue that roots can keep exploring the space unless they are in danger. Light invokes immediate change of root behavior which would facilitate them to grow back into the soil, into darkness [10]. If not illuminated, maize roots keep growing upwards by touching the glass inside surface of the capillary, as shown in the Figure 1. In other words, maize roots can easily grow against the gravity vector if kept in darkness or underground. Root growing within glass capillaries show characteristic the growth pattern with more-or-less spiral trajectory, by repeating touch and release of root tips to the glass surface; little by little, from bottom to the top of the capillary. After emerging out of capillaries, they grow into free space via exploratory-like rhythmic movements resembling crawling or wriggling in the air. Figure 1B depicts the time-lapse pictures showing upside-down maize roots growing inside the glass capillary and emerging out of capillaries into the open space (see the Supplementary movie 1). These findings suggest that tactile information might be important for maize roots to navigate their exploratory growth in such a narrow space.

## Root circumnutation: Exploration of space via spiral movements

Spiral movement is often found in many living organisms. It is thought that this movement has the advantage for exploring broad area efficiently because the trajectories only crosses and do not overlap during this mode of movement. In some insects or birds, such spiral movements have been observed during the search of their nest for homing [11,12]. It is also known that aquatic organisms also have this behavior. For instance, Paramecia, plankton with cilia, normally swim in helical way. Fukui and Asai demonstrated that *Paramecium caudatum* uses tactile sensation obtained by cilia to avoid obstacles during spiral swimming motion in a narrow glass tube with different diameters [13]. Recently, novel 3D tracking system revealed that human sperms also swim helically whereas it was thought to move spirally in 2D plane [14]. This spiraling movement behavior appears to be common strategy for the exploration of available space. Interestingly, also blind persons show this spiral movement when they walk, run, swim, row and drive a car with limited or without eyesight [15]. It was also reported that people getting lost in unfamiliar places (e.g., in dessert or forest) often ended up walking in circles, especially when they could not see the location of sun [16]. Taken together, it might be an intrinsic ability to explore vast area in a similar way in many species.

**Figure 2.**
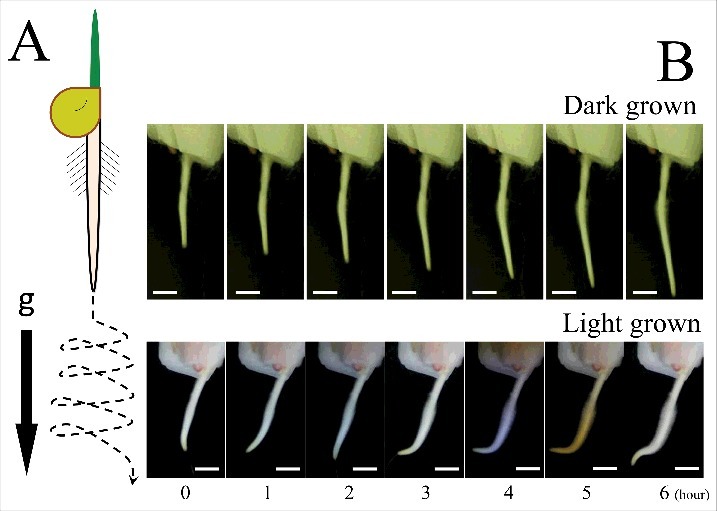
Maize root circumnutation. A. Schematic diagram of root trajectory in exploring movement. B. Roots actively start circumnutating only in light circumstance. Scale bar indicates 1 cm.

Plant organs show spiral movements known as circumnutation [1,17,18]. Figure 2A shows the schematic reconstruction of maize root circumnutation. Root apex actively moves itself and searches the available space. As the Figure 2B shows, this movement is enhanced when roots are grown in the light environment whereas dark-grown roots keep growing toward gravity vector. As discussed above, maize roots inverted in a glass capillary grow upwards in darkness. But, after being illuminated from above, they suddenly accomplished U-turn and started to grow downwards, away from light source [9]. In addition, Arabidopsis roots form skewing and waving spiral root growth pattern on the hard agar under light condition [19,20]. Rice seminal roots grown in light also show wavy patterns [21]. We have previously reported light escape phototropism in Arabidopsis roots [10,22–26]. All this suggest that plant roots change their behavior from pure elongation to exploration mode, namely “circumnutation”, upon illumination or any environmental cues. It is driven by the necessity for positive escape from unfavorable conditions like worm wriggle in danger (see Supplementary movie 2).

## Root cap is essential for exploratory root movements

Since Charles and Francis Darwin performed their de-capping experiments, it is known that intact root caps are essential for root tropisms [1]. Recently, we have discovered that root cap is essential also for the root crawling and U-turn behavior [9]. Root without root cap lacks exploratory movement, crawling behavior [9] Furthermore, auxin molecule, a plant hormone important for plant tropisms, was newly synthesized at root cap and drove U-turn behavior in response to light treatment [27]. Root cap seems to be an important sensor which integrates different environmental information, such as gravity, light, tactile sensation, etc.

## Outlook for root exploration

**Figure 3.**
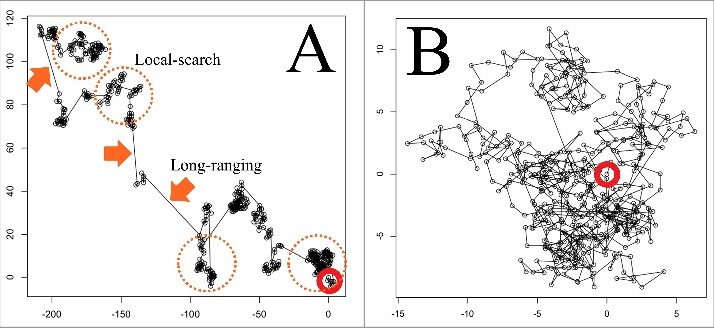
The simulation of Lévy flight model. The plots show the stochastic movement of particle described as small black circle for time step 500. Lines indicate the trajectories of particle movement. A. Lévy flight (α = 1.5), dotted circle indicates the place of local-search. The jumps from place to place as long-ranging are shown. The exponential factor α that controls the tails of power law decay of the Lévy density. B. Ordinary random walk based on Brownian motion (α = 3). Red circle positioned at (0,0) indicates a starting point. The simulation was conducted and plotted with R (version 2.15.2 for Macintosh) software.

Although the movement is controlled by different mechanism in each organism, it is quite intriguing fact that it appears as common behavior in nature for exploring the environments. For robotics, this movement is often mimicked to develop a mapping algorithm for autonomous robots, which explore unknown area [28]. As exploring behavior of foraging animals or insects, several characteristic patterns of action are often found out. The exploration attempts must follow economic principle, i.e., the balance between income and outgo. Extensive and random exploration of environment is energetically costly and thus very expensive. In 1999, “Lévy flight (Lévy walk)” theory based on mathematical random walk with probability distribution was introduced to describe foraging animal behavior, i.e., searching food [29]. The simulation using the model showed repetitive two modes of exploration, local-search and long-range excursions (Figure 3A). This model has been used for evaluating animal behavior because it is more efficient movement for exploring patchy environments than ordinary random walk such as Brownian motion (Figure 3B). Many studies have been confirming that the Lévy walk hypothesis fits to actual exploring/foraging behavior of animals, for example, albatros [30], Drosophila [31], Jellyfish [32] and human [33]. However it is still controversial issue [34]. Nevertheless, this mathematical model might help understanding of the naturally-evolved mode of efficient exploration for natural resources distributed sparsely.

How about plant roots? Exploration of roots in soil can be considered as a foraging behavior for searching nutrient, water, oxygen and space for growth [35,36]. As above described, foraging behavior modeled by Lévy walk consists of two different strategies, local-search and long-ranging (as presented in figure 3A), in many species. Applying an analogy of Lévy walk theory to plant root behavior via tropic movements, these can be considered as long-range excursions and local-search mode, respectively. As we have been discussing above, circumnutation or crawling of roots can be useful for a local-search behavior in soil. The ability of root tips to act as sensory organ, which direct many tropisms, enable for root system to explore quite heterogeneous environments. Plant roots integrate many environmental inputs at the same time as important clues to direct their growth toward favorable circumstances. Besides tactile information, volatile compounds were also shown to affect to root tropism, resembling olfactory sensation of animals [37]. Liu et al. (2018) reported that *C. elegans* nematodes sense odor gradients and steer toward a source of preferred odorant based on a simple nervous circuit [38]. The head-swinging behavior of nematode has a resemblance to that of crawling root behavior. Since it is still very difficult to understand the root behavior as the whole system, the collaborative approach with the field of, for example, animal science, ecology and cognitive science will be necessary to provide new insights in the future.

## Supplementary Material

Movie2.mov

Movie1.mov
